# Incorporating gene expression and environment for genomic prediction in wheat

**DOI:** 10.3389/fpls.2025.1506434

**Published:** 2025-05-06

**Authors:** Jia Liu, Andrew Gock, Kerrie Ramm, Sandra Stops, Tanya Phongkham, Adam Norman, Russell Eastwood, Eric Stone, Shannon Dillon

**Affiliations:** ^1^ Agriculture and Food, Commonwealth Scientific and Industrial Research Organisation (CSIRO), Canberra, ACT, Australia; ^2^ Biology Data Science Institute (BDSI), College of Science, Australian National University, Canberra, ACT, Australia; ^3^ Environment, Commonwealth Scientific and Industrial Research Organisation (CSIRO), Canberra, ACT, Australia; ^4^ Australian Grain Technologies, Roseworthy, SA, Australia

**Keywords:** Bayesian analysis, environmental factor, genomic prediction, omics transcriptome, wheat

## Abstract

**Introduction:**

The adoption of novel molecular strategies such as genomic selection (GS) in crop breeding have been key to maintaining rates of genetic gain through increased efficiency and shortening the cycle of evaluation relative to conventional selection. In the search for improved methodologies that incorporate novel sources of variation for the assessment of genetic merit, GS remains a focus of crop breeding research globally. Here we explored the role transcriptome data could play in enhancing GS in wheat.

**Methods:**

Across 286 wheat lines, we integrated phenotype and multi-omic data from controlled environment and field experiments including ca. 40K single nucleotide polymorphisms (SNP), abundance data for ca. 50K transcripts as well as meta-data (e.g. categorical environments) to predict individual genetic merit for two agronomic traits, flowering time and height. We evaluated the performance of different model scenarios based on linear (GBLUP) and Gaussian/nonlinear (RKHS) regression in the Bayesian analytical framework. These models explored the relative contributions of different combinations of additive genomic (G), transcriptomic (T) and environment (E), with and without considering non-additive epistasis, dominance and genotype by environment (*G* × *E*) random effects.

**Results:**

In controlled environments, where traits were measured under contrasting daylength regimes (long and short days), transcriptome abundance outperformed other random effects when considered independently, while the model combining SNP, environment and *G* × *E* marginally outperformed the transcriptome. The best performing model for prediction of both flowering and height combined all data types, where the GBLUP framework showed slightly better performance overall compared with RKHS across all tests. Under field conditions, we found that models combining all variables were superior using the RKHS framework. However, the relative contribution of the transcriptome was reduced.

**Discussion:**

Our results show there is a predictive advantage to direct inclusion of the transcriptome for genomic evaluation in wheat breeding for traits where *G* × *E* is a factor. However, the complexity and cost of generating transcriptome data are likely to limit its feasibility for commercial breeding at this stage. We demonstrate that combining less costly environmental covariates with conventional genomic data provides a practical alternative with similar gains to the transcriptome when environments are well characterised.

## 1 Introduction

Over the last decade, genomic selection (GS, Meuwissen et al., 2001) has driven significant advancements in animal and plant breeding by allowing breeders to efficiently identify and select individuals with the highest genetic merit in relation to a trait of interest. These approaches work best for highly heritable traits that are complex in their genetic control. GS leverages genomic information to predict the genomic breeding value (GEBVs) of individuals within a population. These predictions are typically derived from high-density single-nucleotide polymorphism (SNP) markers and observed training data using a statistical model. This model fits the relationship between genotypes (SNP) and phenotypic trait(s) based on training population data and partitions the contribution of different genomic effects (e.g., genomic additive and nonadditive effects, including epistasis and dominance) on trait variance. Traditional statistical approaches for GS include genomic best linear unbiased prediction (GBLUP, Clark and van der Werf, 2013) and reproducing kernel Hilbert space regression (RKHS, see, e.g., Gianola and Van Kaam, 2008), a nonlinear Gaussian kernel regression model. GBLUP, based on the linear mixed model, typically uses genomic SNP markers to capture genomic effects from well-defined fixed and random components (Mardia et al., 2024) and is often considered a gold-standard for GS. Due to its nonlinearity, RKHK can effectively capture complex genomic effects from both low- and high-order perspectives, e.g., Heffner et al. (2009).

The potential for other omics data types (e.g., transcriptome, proteome, metabolome) to improve the accuracy of GS in crops has recently gained attention (Li et al., 2019). These biological data layers function between the genome and phenotype expression and can be serve as molecular proxies for phenotype, or endophenotypes (Te Pas et al., 2017). Some studies have demonstrated the value of integrating omics data through prior analysis to elucidate the biological mechanisms driving phenotype variation, thereby informing which genome regions receive greater attention in GS models, such as by weighting gene-based markers (Fang et al., 2017; Ye et al., 2020). This approach relies on detailed experimental work and an in-depth understanding of trait biology and genetics to guide model development, but it carries the risk that biases in interpretation may be propagated in predictions.

A data-driven alternative is to include multi-omics data directly in the predictive framework. Typically, GS is conducted with sparse genomic SNP data, leveraging linkage to capture global genomic variation. Alternate data layers, such as the transcriptome, may offer one avenue to improve the density of biologically relevant markers by focusing on functionally active regions of the genome. It has also been proposed that omics data layers like the transcriptome could improve GS predictions by better capturing computationally elusive epistatic interactions. Computing higher-order interactions among tens of thousands to millions of SNP markers rapidly becomes intractable. The transcriptome provides a biologically informed form of dimensionality reduction, as epistatic interactions among multiple genomic loci may collectively influence transcript abundance (Li et al., 2019). In a mechanistic sense, the transcriptome functions between the genome and phenotype, indirectly capturing both genetics (*G*) and environmental (*E*) effects, as well as their interactions (*G* × *E*). When *G* × *E* significantly mediates trait expression, incorporating transcriptome information provides one avenue to capture these dynamics in prediction frameworks, particularly in cases where environmental effects are poorly characterised.

The majority of studies investigating the utility of transcriptomes in GS have been conducted in maize (Frisch et al., 2009; Fu et al., 2011; Guo et al., 2016; Zenke-Philippi et al., 2017; Xu et al., 2017; Schrag et al., 2018; Westhues et al., 2017; Wang et al., 2019; Azodi et al., 2019). These studies largely illustrate that the transcriptome provides equal or better prediction accuracy than the genome alone. By combining additional omic strata, prediction accuracy can often be improved further, with performance varying slightly depending on the choice of prediction algorithm. However, there are exceptions: for example, Xu et al. (2017) found that genome SNP data were better predictors of yield traits (e.g., ear length, weight) than transcriptome or metabolome data layers in maize. This result potentially points to the opportunistic use of omics data, in which the tissue and the time point for sample collection (immature seed) were not optimised to predict yield traits in the field at maturity. This highlights a significant challenge in implementing large-scale transcriptome studies for trait prediction, where careful factoring of temporal, developmental, and environmental cues in the sampling of endophenotypes is needed. Furthermore, in applied settings, integrating omics data collected in the field for GS will be desirable, though this challenge is exacerbated by greater temporal environmental variability. Despite being a potentially important question to resolve, few studies to date have explored the use of endophenotypes under field conditions for GS applications, including feasibility in commercial breeding programs, suggesting that more work is needed across a broader range of crops. While numerous studies have focused on maize, none have examined wheat. Significantly, the application of multi-omics for GS in wheat, an important staple crop in Australia and globally, has yet to be explored.

Wheat productivity has been maximised by optimising flowering time to match local climates through genetic and environmental selection (Reynolds et al., 2012; Hyles et al., 2020). This involves understanding genetic and environmental (*G* × *E*) interactions, some of which are well-characterised (Crossa et al., 2021; Tolhurst et al., 2022). Flowering time in wheat could serve as a model for studying genomic, endophenomic, and environmental influences on trait variation. As a deterministic trait, its molecular regulation is established early in development, with detectable expression patterns before floral transition (VanGessel et al., 2022; Shi et al., 2019). This makes flowering a valuable platform for exploring whether early-stage endophenotypes can predict later developmental traits, facilitating breeding efficiency. Environments experienced at all growth stages can shift the variation in phenology via *G* × *E*. Thus, another important question is the extent to which such variation impacts the efficacy of endophenotypes for GS in the field relative to static conditions in controlled environments. This will be particularly relevant for broad-acre, dryland crops such as wheat. Lastly, given that endophenotypes are an expression of underlying genetics and environment, something that has received less attention in the literature is whether they can be more efficiently represented by robustly capturing *G*, epistasis, and *G* × *E* interactions in prediction frameworks.

This study evaluates two widely used genomic selection approaches to predict agronomic traits in wheat using a diverse wheat panel and data collected in both field and controlled environments. Specifically, we use a linear mixed model, GBLUP, as a benchmark to compare with a nonlinear Gaussian kernel regression (RKHS) under the Bayesian framework and compare different model scenarios designed to test the relative merits of applying different combinations of predictor variables: additive genomic (*G*), transcriptomic (*T*), and environmental (*E*) covariates, with and without considering nonadditive random genomic effects (epistasis and dominant) and *G* × *E*. We highlight the value of including transcriptome for prediction and its potential for application in wheat breeding. We also consider challenges associated with field-based transcriptome-wide experiments, including the importance of choosing the appropriate endophenotype tissue and timing of sampling in settings where the environment changes throughout plant development. Considering these limitations and the greater cost of data generation, we ask whether the inclusion of the transcriptome is feasible for commercial breeding and explore the role of lower-cost alternatives, such as including *G* × *E*, in supporting improvements for GS.

## 2 Materials and methods

### 2.1 Data and experiments

This study used the OzWheat diversity panel, a collection of 286 wheat lines that includes land races and progenitors of early Australian varieties, additional founders that emerged through the Green Revolution, and a larger number of modern Australian elite varieties (Hyles et al., 2024; Dillon et al., 2025). The 286 selected wheat lines (Triticum aestivum) from the whole panel (ca. 600 lines) were used in controlled environments. Across field experiments, there were slight variations in which of the 286 lines were used, depending on the quantities of seed available at the time of sowing.

#### 2.1.1 Controlled environment experiments

All data collected in controlled environments were reported in detail by Dillon et al. (2025). In brief, panel genotypes were grown under contrasting “long” (16 h light) and “short” (8 h light) photoperiods in controlled-environment growth chambers (PGC20 Conviron^®^, Winnipeg, Canada). For each variety, there were six biological replicates in a randomised complete block experiment design, all of which were analysed. Panel genotypes were also grown under contrasting “long” (12 h light) and “short” (8 h light) photoperiods in a double-coated plastic growth house with temperature control in Canberra.

##### 2.1.1.1 Trait data

Plants were subject to twice-weekly assessments to detect flowering. This was based on plants having reached stage Z61 (the Zadoks stage, Zadoks et al., 1974), or “anthesis”, measured in days after sowing and marked by the extrusion of anthers from the spikelets. The height (cm) of each plant was measured at maturity and included the total above-ground stem length plus the total spike length. Both of these traits are highly heritable with strong genetic control and, in the case of flowering, exhibit strong environmental and *G* × *E* interactions with photoperiod variation.

##### 2.1.1.2 Transcriptome data

The crown plus coleoptile was harvested at the two-leaf stage (Z12) from all seedlings grown in the cabinet experiment, which were immediately stored in prelabelled tubes and frozen in liquid nitrogen. Samples were subsequently transferred to − 80°C for long-term storage. Sample collection was timed to occur over the 2 h leading up to midday in each treatment (long and short day lengths). Cabinet time of day was staggered by 2 h between treatments to allow for both treatments to be sampled on the same day. Ribonucleic acid (RNA) was extracted from entire frozen tissue sampled from a single biological replicate of each panel variety, and libraries were prepared for RNA-seq. Sequenced reads were quality-checked, trimmed, and mapped against the Chinese Spring reference coding sequence v1.0 using the Trinity package (Haas et al., 2013), for the estimation of expression abundance for 44,054 coding genes.

##### 2.1.1.3 Genome SNP data

SNP data were obtained from two sources. Trimmed paired-end sequence reads for each sample were merged across treatments and aligned to the Chinese Spring coding sequence (CDS) reference v1.0 (IWGSC, 2018) using BWA-MEM (Li, 2013, settings), and SNP variants were called using GATK3.7 haplotype caller as described by Dillon et al. (2025), yielding ca. 12,000 SNP markers. These were combined with ca. 21,000 SNPs from the 90,000 Illumina Infinium SNP array (Wang et al., 2014) to make up a total set of 33,174 SNP markers for downstream analysis.

#### 2.1.2 Field experiments

**Figure 1:**
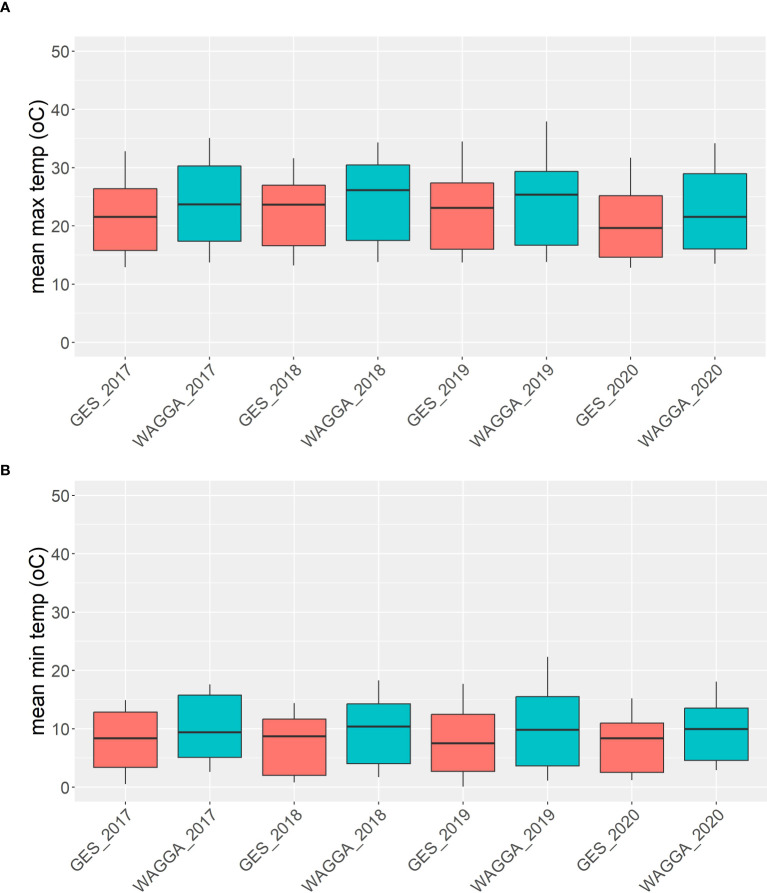
Means of maximum **(A)** and minimum **(B)** temperatures from five different field trials in Canberra and Wagga Wagga over four different years between 2017 and 2020.

In total four field experiments were conducted at the CSIRO Ginninderra Experimental Station (GES) near Canberra in 2018 (35°11′59″S 149°04′48″E) and 2019 (35°10′58″S 149°03′30″E), and at the Australian Grain Technologies (AGT) breeding site, Kabinga near Wagga Wagga in 2018 (35°03′28″S 147°02′44″E) and 2019 (35°03′29″S 147°02′51″E), as previously described by Hyles et al. (2024). Two replicates of each line were sown in a randomised complete block design at each site (*n* = 260 at Wagga, *n* = 280 in Canberra). Experiments were sown at GES on the 5th of June and 30th of April, and on the 25th of May and 28th of May at Kabinga in 2018 and 2019, respectively. In Canberra, each plot comprised eight rows with 18 cm spacing and a length of five linear metres. At Wagga Wagga, plots comprised two rows only, with total plot dimensions of 0.75 linear metre × 2.5 linear metres. Environmental covariates were not used to characterise experiments in our analysis; rather, we treated the site as a categorical variable in our models. Nevertheless, environmental conditions varied significantly between our chosen locations. Canberra was consistently cooler throughout the growing season compared to Wagga Wagga during the years our experiments were conducted, as shown in **Figure 1**. The sites are similar in terms of latitude and, hence, photoperiod during the growing season.

##### 2.1.2.1 Trait data

For each plot, we obtained estimates of the date to heading (Z51, the date when 50% of plants in the plot had spikes fully emerged from the boot), with the exception of Wagga Wagga 2019, where the heading date was only obtained from a single replicate block. Height (cm) at maturity was measured for three representative plants per plot and included the total aboveground stem length and the total spike length. Both traits are highly heritable with strong genetic control, and in the case of flowering, exhibit strong *G* × *E* interactions with thermal and vernal accumulation. The same technique for trait data collection was used as described by Hyles et al. (2024).

##### 2.1.2.2 Transcriptome data

The crown plus coleoptile was harvested at the Z12 from two representative seedlings per field plot in the first block. The samples were immediately stored in prelabelled tubes, frozen in liquid nitrogen, and stored on dry ice for transport back to the laboratory. They were subsequently transferred to − 80°C for long-term storage. Sample collection was timed to occur over the 2 h leading up to midday. RNA was extracted from frozen tissue using the Maxwell^®^ RSC Plant automated extraction system following the manufacturer’s instructions (Promega, Australia, catalogue number AS1500), and quality was checked according to the method described above. RNA libraries were generated using the method of Wang et al. (2014) with modifications. The same method was used to collect the transcriptome data as described in Dillon et al. (2025). The multiplexing design used 384 polymerase chain reaction (PCR) primer combinations to introduce the dual-end 8-bp index sequence to the final library product using the TruSeq backbone, which was compatible with the Illumina Novaseq 6000 sequencing platform. Libraries from each experiment were sequenced on one lane of a Novaseq 6000 S4 flow cell. Using the same workflow as described above for the controlled environment experiments, the abundance of 70,606 coding genes was obtained from the sequence data and represented as a sparse matrix for downstream analysis. The SNP data used in combination with the field trait data are the same as those described above for the controlled environment experiments.

### 2.2 Statistical models for genomic selection and prediction

#### 2.2.1 GBLUP

The conventional genomic best linear unbiased prediction (GBLUP) model only considers the genomic additive random effect, additive random effect with a simple expression as follows:


(1) y=μ1+ɡ+ϵ,

where y is the trait of interest, μ is a n×1 vector that describes the fixed effect, the noise term $\text{ϵ}∼N\left(0,I_{l\times l}\sigma_{\text{ϵ}}^{2}\right)$ . We have the genomic random effect $\text{ɡ}={\left({ɡ}_{1},{ɡ}_{2},\cdots ,{ɡ}_{l}\right)}^{T}∼N\left(0,\sigma_{ɡ}^{2}G\right)$ , which follows a multivariate Gaussian distribution (MVN) with zero mean and a covariance matrix defined as the product of genomic variance $\sigma_{ɡ}^{2}$ and the genomic relationship matrix (GRM), *G* (VanRaden, 2008; Casella and Berger, 2024), representing the covariance between pairwise wheat lines (genotype). We use a method proposed by Endelman and Jannink (2012) to estimate GRM via the linear kernel, $G=\frac{WW^{T}}{2\sum_{k}p_{k}\left(1-p_{k}\right)}$ where *W* is the cantered genotype matrix, with *W_ik_* = *X_ik_* − 2*p_k_*, *k* = 1,2,···,*m*. The matrix *X* is a *l* × *m* matrix containing genomic SNP markers for *l* individual lines and *m* biallelic (AA, AB, and BB) SNP markers coded as [−1,0,1] or [0,1,2] for each line. The allele frequency *p_k_* is given by $p_{k}={\left(2l\right)}^{\left(-1\right)}\sum_{i=1}^{l}X_{ik}$ and is calculated from DNA SNP sequences for each individual line (see Müller et al., 2015). This setting ensures that the proper scaling of the diagonal element of the estimated GAM is equal to 1 + *f*, where *f* is the inbreeding coefficient of the current population of interest. When n≠l , meaning the number of trait observations does not match the number of individual lines, an incidence matrix *Z_g_* (Li et al., 2019) (0 absence and 1 presence) can be introduced, such that $\text{g}∼N\left(0,\sigma_{g}^{2}Z_{g}GZ_{g}^{T}\right)$ , to ensure that the genotypes relate to the phenotype observations. For example, suppose *G* is a 3 × 3 GRM that describes correlations between three individual lines, and we have 10 trait observations: five from the first line, three from the second line, and two from the third line, then we have an incidence matrix $Z_{g}^{T}$ with the dimension 3 × 10 given by


$$Z_{ɡ}^{T}=\;\;\;\;\begin{array}{cccccccccc} 1 & 1 & 1 & 1 & 1 & 0 & 0 & 0 & 0 & 0\\ 0 & 0 & 0 & 0 & 0 & 1 & 1 & 1 & 0 & 0\\ 0 & 0 & 0 & 0 & 0 & 0 & 0 & 0 & 1 & 1 \end{array}\;.$$

Nonadditive genomic random effects include epistasis (or gene-by-gene, *G* # *G*) and dominant (*A*). Epistasis refers to gene–gene interactions between loci and can appear in biallelic and/or high orders. Epistasis has been reported to modify phenotypic traits in crops and may offer advantages for GS, as noted by Doust et al. (2014). In contrast, the dominant effect describes interactions between alleles at the same locus and typically refers to heterozygous alleles (AB). To analyse the dominant effect of the biallelic SNP markers, we can encode the SNP markers as 1 (AA, BB) and 0 (AB).

To integrate nonadditive genomic random effects with the additive, the traditional GBLUP model in Equation 1 can be extended by


(2) $$\text{y}=\mu 1+\text{g}+{\text{g}}_{\mathrm{ep}}+{\text{g}}_{a}+\text{ϵ},$$

Where $g_{1}$ is the genetic additive random effects as g in Equation 1, $g_{2}$ stands for the epistasis, and ${\text{g}}_{a}$ represents the dominant effect. Due to the property of epsitasis, we can assume ${\text{g}}_{\mathrm{ep}}$ follows a Gaussian distribution with zero means and a product of the linear kernel *G*, that is, $\text{g}∼N\left(0,\sigma_{ep}^{2}G\#G\right)$ , where *G* # *G* denotes the epistatic relationship matrix calculated using the Hadamard product between the GRM (Henderson, 1985; Jiang and Reif, 2015), and $\sigma_{ep}^{2}$ is the epistasis variance. The dominant random effect $g_{a}∼N\left(0,\sigma_{a}^{2}A\right)$ follows a MNV with zero means and covariance derived from the dominant relation matrix *A* and the variance $\sigma_{a}^{2}$ . If n≠l , the same incidence matrix *Z_g_* can be introduced to each random effect in Equation 2.

Li et al. (2019) extended the GBLUP model to a GTBLUP model in Equation 3 by integrating omics transcriptome (*T*) data using the mathematical model below


(3) y=μ1+g+t+ϵ,

Where the transcriptome effect is indicated by *t*, which can be assumed to follow a MVN, $t∼N\left(0,\sigma_{t}^{2}Z_{t}TZ_{t}^{T}\right)$ as *g*, *Z_t_* is an incidence matrix. The corresponding *n* × *n* variance–covariance matrix *T* is a linear kernel calculated from normalised transcriptome data.

The influence of gene-by-environment (*G* × *E*) interactions in crops has been studied in recent decades (Doust et al., 2014; Jarquin et al., 2014; Bandeira e Sousa et al., 2017; Lopez-Cruz et al., 2015). *G* × *E* interactions refer to certain situations in which the effects of a relative allele vary across environments. It has been claimed to have a strong effect on some visible traits, such as branching and seed size (Sadras and Slafer, 2012). In this paper, we analyse two agronomic traits in wheat—height and flowering time—that are affected by multiple random effects introduced earlier. We thus extend the model in Equation 3 by incorporating *G* × *E*,


(4) y=μ1+g+t+gE+ϵ,

Where gE is the random effect of GxE interactions and $gE∼N\left(0,{\text{σ}}_{gE}^{2}Z_{g}GZ_{g}^{T}\circ Z_{E}Z_{E}^{T}\right)$ , where *Z_E_* is the incidence matrix for the effects of environments on the traits, and ∘ denotes the Hadamard or Schur product, which describes the element-to-element product between two matrices of the same order (Bandeira e Sousa et al., 2017; Jarquin et al., 2014). Additionally, ${\text{σ}}_{gE}^{2}$ is a vector of J variance components of *G* × *E* interactions, and each ${\text{σ}}^{2}\left\{gE\_j\right\}$ in ${\text{σ}}^{2}\left\{gE\right\}$ indicates the *G* × *E* variance for *j*th environment.

##### 2.2.1.1 Modelling G X E

Equations 1–3 described the regression of a single environment. When extending to multiple environments, we introduce the foot index *j* so that in the *j*th environmental stratification, the model in Equation 4 becomes


(5) $${\text{y}}_{j}={\text{μ}}_{j}1+{\text{g}}_{j}+t_{j}+{\left(g\text{E}\right)}_{j}+{\text{ϵ}}_{j}.$$

The observed trait data in Equation 4 is structured from all *j* environments from Equation 5, which is given by


$$\text{y}:=[\begin{array}{c} {\text{y}}_{1}\\ \vdots \\ {\text{y}}_{j}\\ \vdots \\ {\text{y}}_{J} \end{array}].$$

We apply the multi-environment single-variance model proposed by Bandeira e Sousa et al. (2017) to capture the *G* × *E* interactions by


(6) $$Z_{ɡ}GZ_{ɡ}^{T}\circ Z_{E}Z_{E}^{T}=\left\lceil \begin{array}{ccccc} G_{1} & \cdots & 0 & \cdots & 0\\ \vdots & \ddots & \vdots & \ddots & \vdots \\ 0 & \cdots & G_{j} & 0 & \cdots \\ \vdots & \ddots & \vdots & \ddots & \vdots \\ 0 & \cdots & 0 & \cdots & G_{j} \end{array}\right\rceil \;,$$

Where *G_j_* represents correlations between wheat lines in the *j*th environment. The main reason for applying this model is that our data were collected under environmental scenarios. Other possible *G* × *E* models can be found in, e.g., Jarquin et al. (2014); Bandeira e Sousa et al. (2017), and Lopez-Cruz et al. (2015).

#### 2.2.2 RKHS

RKHS, as introduced by Berlinet and Thomas-Agnan (2004), defines a kernel function: k : X×X→ℝ named a reproducing kernel over a nonempty feature set X through a map ϕ : X →ℋ over a Hilbert space ℋ such that:


$$\begin{array}{l} \forall x\in X,k\left(x,x^{\prime }\right),\in H\\ \forall x\in X,\forall \phi \in ℋ,<\phi ,k(.,x){>}_{ℋ}=\phi \left(x\right). \end{array}$$

In genomic prediction, the RKHS model introduces a nonlinear Gaussian kernel on the SNP marker matrix, $K\left(X,X^{\prime }\right)=\text{exp }(-\frac{{\left(X-X^{\prime }\right)}^{2}}{h})$ (Li et al., 2019; Jarquin et al., 2014; Costa-Neto et al., 2021) to capture a mixed genomic random effect that includes additive effects as well as complex cryptic interactions, which we refer to as *epistasis*, between pairwise SNP markers, along with dominant effect (*A*). This represents a major difference between the RKHS model and the GBLUP models, where a linear kernel is applied in GBLUP to capture additive (GAM) and nonadditive (epistasis and dominant) genomic random effects separately. Additionally, this nonlinear kernel can be used to describe other random effects in the model. The parameter *h* in the above Gaussian kernel describes a bandwidth parameter that controls the decay rate of correlations between individuals.

#### 2.2.3 Bayesian inference for prediction

##### 2.2.3.1 Bayesian theorem and the hierarchical model

Let **u** represent the unknown random effects of interest in the model, and denote the corresponding variance as ${\text{σ}}_{u}^{2}$ . We assume ${\text{σ}}_{\text{ϵ}}^{2}$ = {*µ,σ_u_,θ,σ_Є_*}, where *θ* includes all possible hyperparameters in the kernel functions. The likelihood can then be expressed as


(7) $$p(y|u,\text{ξ})=\prod_{i=1}^{n}N(y_{i}|u_{i},\text{ξ})$$

By the Bayesian theorem (Gelman et al., 1995), the joint posterior can be approximated by the product of the likelihood in Equation 7 and the prior defined in Equation 9, and is given by


(8) $$p(u,\xi |y)\propto \prod_{i=1}^{n}N(y_{i}|u_{i},\xi )N(u_{i}|0,\text{K}\sigma_{u})\prod_{k=1}^{q}p\left(\xi_{k}\right)$$

Where *N*(*u_i_* |0, ***K*** *σ_u_*) is the Gaussian prior of the unknown random effects. The variance–covariance matrix ***K*** captures the correlations from different types of input data. The hyperprior density of the hyperparameter ***ξ*** can be expressed by


(9) $$p\left(\xi \right)=\prod_{k=1}^{q}p\left(\xi_{k}\right)=p\left(\mu \right)p\left(\sigma_{Є}\right)p\left(\sigma_{u}\right)\prod_{k=1}^{q-3}p\left(\theta_{k}\right).$$

We optimise the model in Equation 8 by maximising the logarithmic posterior up to a constant w.r.t. ***ξ***,


(10) $$\text{arg }\underset{\xi }{\max}\left(\sum_{i}\text{log }p(y_{i}|u_{i},\xi )+\sum_{k}\text{log }p\left(\xi_{k}\right)\right).$$

We then use the posterior predictive density, which follows a Gaussian distribution from the trained model with the optimal value $\hat{\xi }$ to do the prediction at new input data *X* ^∗^,


(11) $$p(u\left(X^{*}\right)|y,u\left(X\right),\hat{\xi })$$

By choosing conjugate priors for the model parameters, e.g. *p*(***µ***) is constant, *p*(***u***) follows the multivariate Gaussian distributions, *p*(*σ_ϵ_*) and *p*(*σ_u_*) follow the scaled inverted *χ* ^2^ distributions (see Chapter 16 of Mrode, 2014 for more details), we can obtain close forms of the full conditional posterior distributions of each parameter and hyperparameter of interest. Predictive values at new input data can then be computed from the posterior predictive distribution in Equation 11; see Rasmussen (2006) for reference. There are multiple ways to train the model. In this work, we apply the Gibbs sampler through the Markov chain Monte Carlo (MCMC) method to optimise ***ξ***; see Robert and Casella (2005) for more details.

##### 2.2.3.2 The eigen-decomposition transformation

The eigen-decomposition is widely used in computations to ensure stability and efficiency by maintaining a well-conditioned and symmetric variance–covariance matrix. Through the eigen-decomposition, we obtain


(12) $$K=U\Lambda U^{T},$$

where *U* is a *n* × *n* square matrix whose *i*th column is the corresponding eigenvector of ***K***, and *U* is orthogonal such that *UU^T^* = *I_n_*. The elements in the diagonal matrix **Λ** are the eigenvalues of ***K***.

By the eigen-decomposition transformation, we have a new random vector $s:=U^{T}u∼N\left(0,\text{Λ}\sigma_{u}^{2}\right)$ such that **Λ** = *U^T^ **K**U*. This transformation immediately results in the likelihood in Equation 7 to be:


(13) $$p(t|s,\xi )=\prod_{i=1}^{n}N(t_{i}|s_{i},\xi ),$$

Where $t:=U^{T}y$ . In other words, when updating *ξ* in Equation 10 by replacing the likelihood by Equation 13, we only need to use a diagonal matrix **Λ** in Equation 12 with eigenvalues from ***K*** instead of referencing the variance–covariance matrix ***K*** in MCMC or alternative methods.

##### 2.2.3.3 GEBV

GEBV refer to the sum of all breeding values at each locus (Mrode, 2014). They can be approximated by the posterior mean and posterior predictive mean of genetic random effects in Equations 1 or 2 for the training and test populations, respectively.

#### 2.2.4 Model scenarios

We examined the value of different explanatory variables—genomic (*G*), transcriptomic (*T*), environment (*E*)—their interactions (*G* × *E*, *G* # *G*), and alternate model frameworks (linear/nonlinear) for predicting flowering time and height, structuring our analyses across 13 model scenarios. The traditional GBLUP model was applied as our benchmark as follows:

##### 2.2.4.1 Models 1–3, additive genomic and nonadditive genomic random effects (epistasis and dominance)


(14) y=μ1+g+є, (G)

(15) $$y=\mu 1+g+g_{\mathrm{ep}}+є,\;(G+G\#G)$$

(16) $$y=\mu 1+g+g_{\mathrm{ep}}+g_{a}+є,\;(G+G\#G+A)$$

##### 2.2.4.2 Models 4–5, additive genomic plus genomic and environmental random effects


(17) y=μ1+g+gE+є, (G+G×E)

(18) $$y=\mu 1+g+gE+g_{\mathrm{ep}}+\text{є},\;(G+G\times E+G\#G)$$

##### 2.2.4.3 Models 6–7, additive transcriptomic and genomic plus nonadditive genomic random effects


(19) y=μ1+g+t+є, (G+T)

(20) $$y=\mu 1+g+t+g_{\mathrm{ep}}+є,\;(G+T+G\#G)$$

##### 2.2.4.4 Models 8–9, plus all interaction between omic and environmental random effects


(21) $$y=\mu 1+g+t+gE+g_{\mathrm{ep}}+є,\;(G+T+G\times E+G\#G)$$

(22) $$y=\mu 1+g+t+gE+g_{\mathrm{ep}}+g_{a}+є,\;(G+T+G\times E+G\#G+A\text{).}$$

Due to the nonlinearity of the Gaussian kernel, genomic additive and nonadditive random effects are captured together with *G* ^∗^, which includes *G*, epistasis (EPI, *G* # *G*), and dominant (*A*). Therefore, we only compare the following RHKS models with the GBLUP benchmark:

##### 2.2.4.5 Models 10–11, genomic and transcriptome random effects and their interaction


(23) $$y=\mu 1+g^{*}+є,\text{ (}G^{*})$$

(24) y=μ1+t+є, (T)

(25) $$y=\mu 1+g^{*}+t+є,\text{ (}G^{*}+T)$$

##### 2.2.4.6 Model 12, plus environmental interactions


(26) $$y=\mu 1+g^{*}+t+g^{*}E+є,\text{ (}G^{*}+T+G\times E)$$

#### 2.2.5 Model validation and assessment

We evaluated the predictive accuracy of the model by fivefold crossvalidation (CV, e.g., Allen, 1974) because the ground truth is unknown. This involved randomly partitioning the whole population into five equal-sized subsamples. In the CV procedure, each subsample, representing around 20% of the test population from raw data of each environment, was used to validate the respective model splits trained on the remaining 80% of the population. This process was repeated five times. For single-environment models, stratification analysis (Lopez-Cruz et al., 2015) was applied, and the mean predictive accuracy was computed between two environments. The environments from field experiments were represented by two site indicators (GES and Wagga). Due to the balanced data in the experiments and the simple categorical environmental variables, this validation scenario was used for model comparison in both controlled and field experiments. In addition, height phenotypic values from field experiments were collected three times, with independent analyses carried out, and the mean performance demonstrated. Overall model accuracy was obtained by averaging the fivefold model accuracies. Additional validation scenarios, such as CV1 and CV2 (Alemu et al., 2024), may be necessary to evaluate model performance across different environmental conditions; however, this is beyond the scope of the present study.

For model assessment, we compute Pearson correlation *r* using Equation 27 between the true trait values, $y_{t}\;$ from the test population and the predictive traits $\hat{y_{t}}$ from the predictive model. This ratio measure provides a statistic for model predictive accuracy.

Despite training and test populations, the general formula of Pearson correlation is given by


(27) $$r=\frac{\sum_{i}\left(y_{i}-\bar{y})({\hat{y}}_{i}-\bar{\hat{y}}\right)}{\;\|y_{i}-\bar{y}\|\left\|{\hat{y}}_{i}-\bar{\hat{y}}\right\|}.$$

In this work, we study the impacts of genomic additive and nonadditive random effects, omics data, and *G* × *E* interactions on phenotypic traits over two different day lengths to improve GP and GS in wheat traits. We construct a Bayesian hierarchical model by estimating the parameters of interest in order to compare the predictive models listed in Sect. 4.2. We chose a Gaussian prior for the unknown random effects ***u*** and conjugate priors for ***ξ***, specifically assigning the scaled inverse *χ* ^2^ distribution to all the variance parameters, with hyperparameters set to fixed values from the R software package, Bayesian generalised linear regression (BGLR; see more details in Pérez and De Los Campos, 2014). The aforementioned models were trained by optimising the unknown parameters using the MCMC method, specifically by simulating each parameter using the Gibbs sampler from its full conditional posterior distribution formed by the likelihood in Equation 13 after the eigen-decomposition transformation and the selected prior. We also utilised the R software package BGGE to compute *G* × *E* interactions in Equation 6. All of our work was implemented using the R software.

## 3 Results

**Figure 2:**
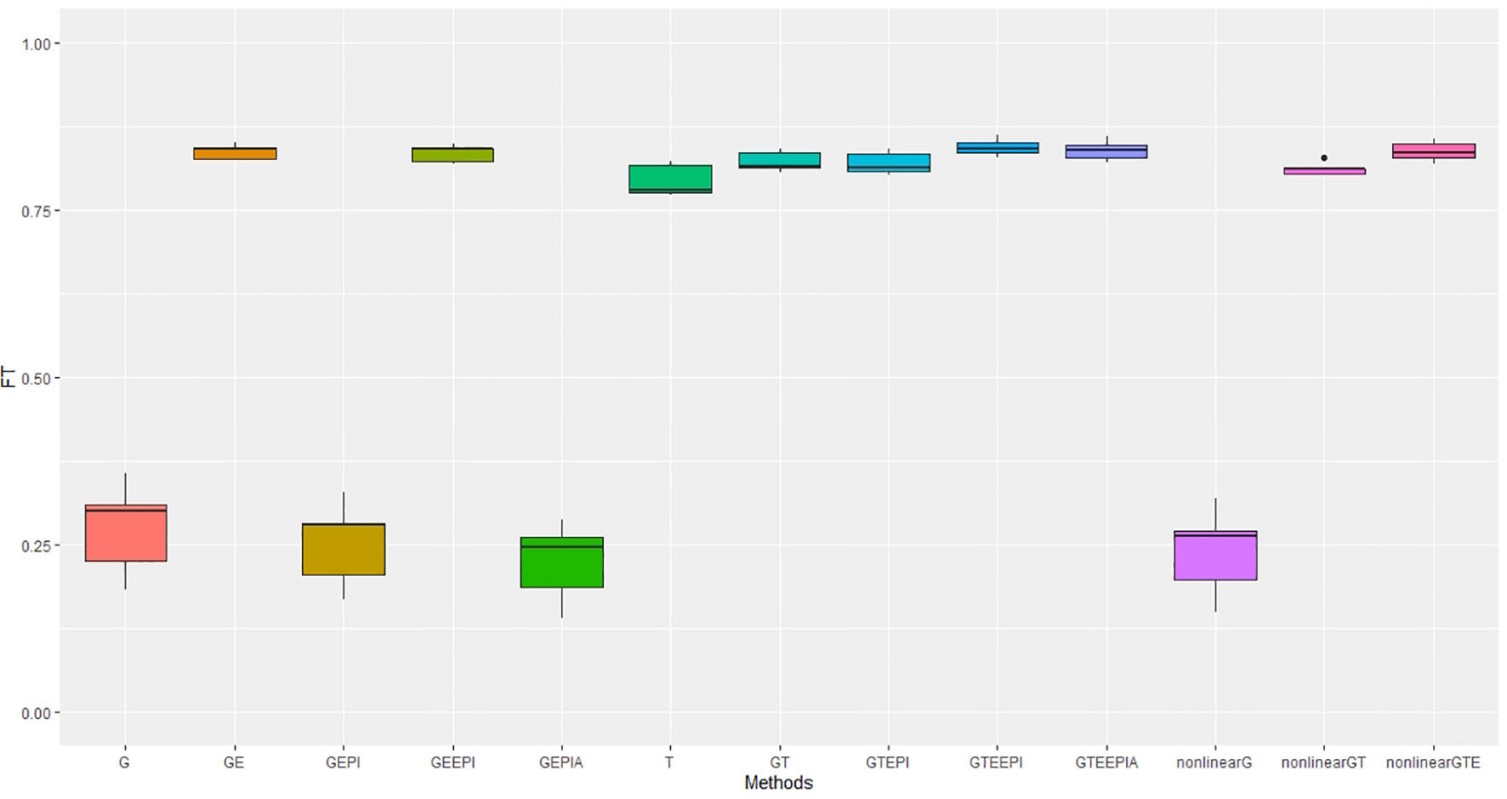
Performance metrics (Pearson correlation) for genomic predictive accuracy on FT across two regression models: GBLUP and RKHS (nonlinear) from controlled environmental data. Error bars represent model performance among 13 different model scenarios. The *x*-axis provides abbreviated model names consistent with the detailed scenarios described: GBLUP: G, G + G × E (GE), G + G # G (EPI) + A (dominant) (GEPIA), G + G × E + G # G (GEEPI), G + T (GT), G + T + G # G (GTEPI), G + T + G × E + G # G (GTEEPI), G + T + G × E + G # G + A (GTEEPIA); RHKS: G + G # G + A (nonlinearG), T (T), G + G # G + A + T (nonlinearGT), G + G # G + A + T + G × E (nonlinearGTE).

**Figure 3:**
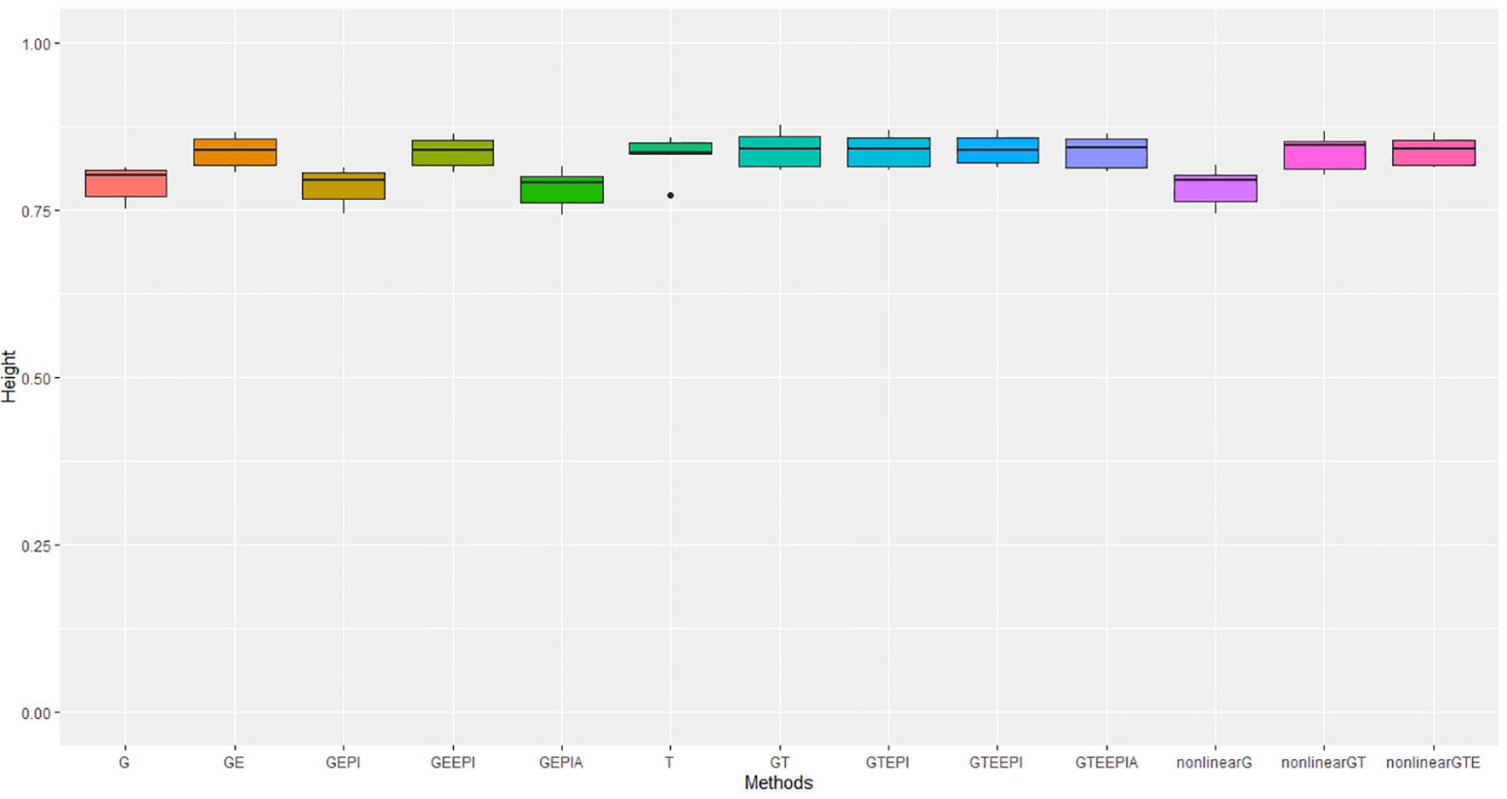
Performance metrics (Pearson correlation) for genomic predictive accuracy on height across two regression models: GBLUP and RKHS (nonlinear) from controlled environmental data. Error bars represent model performance among 13 different model scenarios. The *x*-axis provides abbreviated model names consistent with the detailed scenarios described: GBLUP: G, G + G × E (GE), G + G # G (EPI) + A (dominant) (GEPIA), G + G × E + G # G (GEEPI), G + T (GT), G + T + G # G (GTEPI), G + T + G × E + G # G (GTEEPI), G + T + G × E + G # G + A (GTEEPIA); RHKS: G + G # G + A (nonlinearG), T (T), G + G # G + A + T (nonlinearGT), G + G # G + A + T + G × E (nonlinearGTE).

In controlled environments (**Figures 2**, **3**), where traits were measured under contrasting long and short day length regimes, transcriptome abundance (*T*) in Equations 19, 20 outperformed genomic SNPs (*G*) in Equations Equations 14-16 when they were modelled without environmental factors in the GBLUP regression framework. The improvement was more pronounced for flowering time than for height, supporting the hypothesis that the transcriptome, as an intermediate state between genome, environment, and final phenotype, is a good predictor of trait variation. In our study, models combining SNP data and *G* × *E* effects marginally outperformed transcriptome for both traits. The best-performing models combined all data types—*G* × *E* interactions, and epistasis—with both the GBLUP and RHKS frameworks. Notably, GBLUP demonstrated slightly better overall performance compared to RHKS in all tests. This finding aligns with previous research indicating that GBLUP often provides higher predictive accuracy than RKHS, particularly for traits where additive genetic effects are predominant.

In terms of nonadditive interaction effects, the inclusion of *G* × *E* had the most positive impact on prediction accuracy, particularly for flowering time. Prediction of height was less dependent on interactions with the environment; hence, the *G* and *G* + G × *E* models (Equations 14, 17) performed better relative to anthesis. Explicitly fitting epistatic (EPI) and dominance (*A*) interactions based on SNP covariance slightly reduced model accuracy under the GBLUP for both agronomic traits. While we cannot test this trend precisely with RHKS because *G* and *G* # *G* cannot be disentangled, the RHKS model scenarios (Equation 23) including nonadditivity outperformed their GBLUP equivalents (Equation 16) in field experiments.

**Figure 4:**
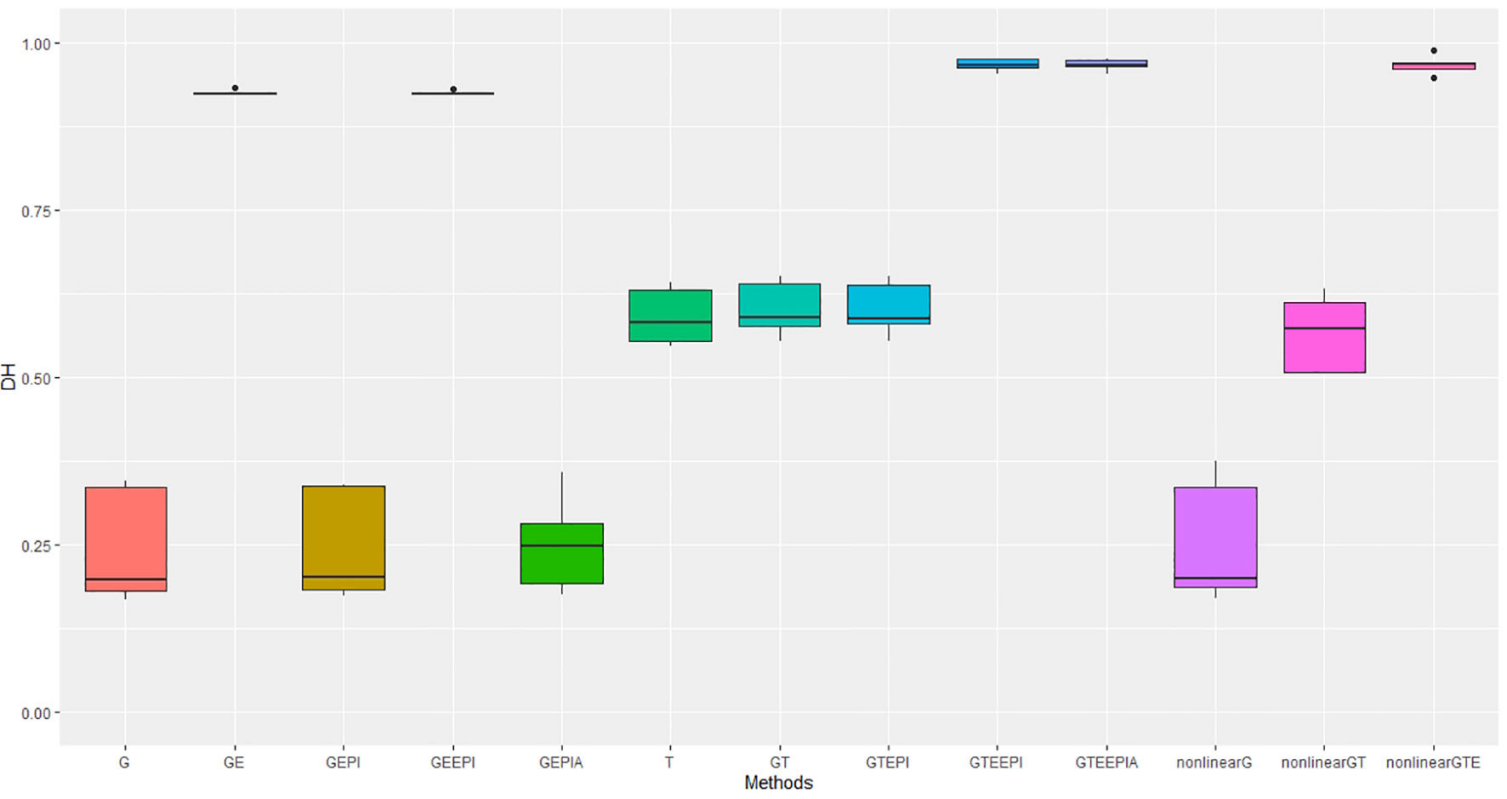
Performance metrics (Pearson correlation) for genomic predictive accuracy on DH across two regression models: GBLUP and RKHS (nonlinear) from field experiment data. Error bars represent model performance among 13 different model scenarios. The *x*-axis provides abbreviated model names consistent with the detailed scenarios described: GBLUP: G, G + G × E (GE), G + G # G (EPI) + A (dominant) (GEPIA), G + G × E + G # G (GEEPI), G + T (GT), G + T + G # G (GTEPI), G + T + G × E + G # G (GTEEPI), G + T + G × E + G # G + A (GTEEPIA); RHKS: G + G # G + A (nonlinearG), T (T), G + G # G + A + T (nonlinearGT), G + G # G + A + T + G × E (nonlinearGTE).

**Figure 5:**
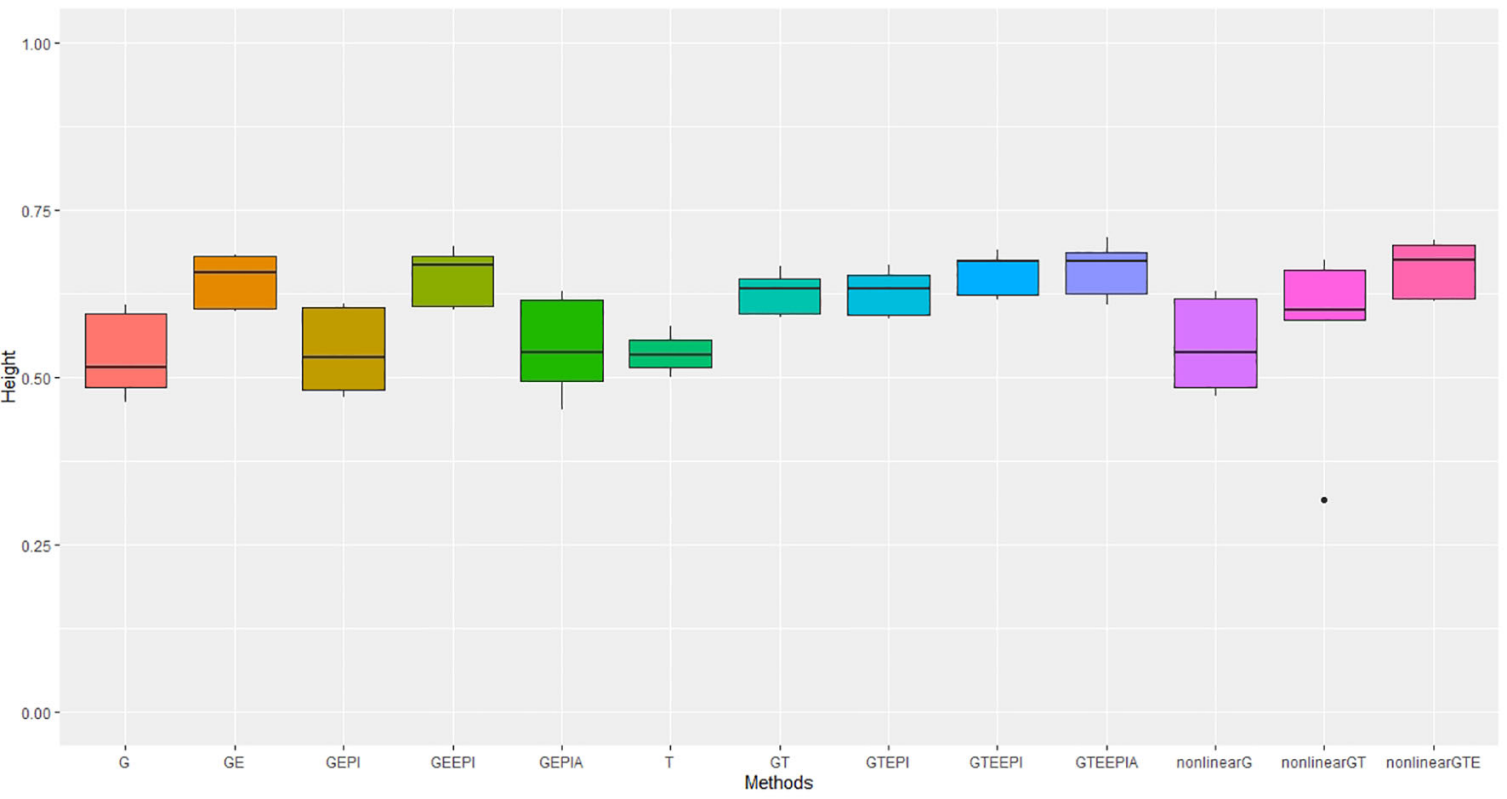
Performance metrics (Pearson correlation) for genomic predictive accuracy on height across two regression models: GBLUP and RKHS (nonlinear) from field experiment data. Error bars represent model performance among 13 different model scenarios. The *x*-axis provides abbreviated model names consistent with the detailed scenarios described: GBLUP: G, G + G × E (GE), G + G # G (EPI) + A (dominant) (GEPIA), G + G × E + G # G (GEEPI), G + T (GT), G + T + G # G (GTEPI), G + T + G × E + G # G (GTEEPI), G + T + G × E + G # G + A (GTEEPIA); RHKS: G + G # G + A (nonlinearG), T (T), G + G # G + A + T (nonlinearGT), G + G # G+ A + T + G × E (nonlinearGTE).

Predictive accuracy for height and days to heading measured in four field experiments over 2 years suggests similar outcomes for the tested model scenarios as observed in controlled environments, with the following exceptions. Under field conditions, (**Figures 4**, **5**) the predictive accuracies were lower for height across all model scenarios, whereas predictions for flowering for the best-performing scenarios were higher than in controlled environments. In particular, the improvement gained from including *G* × *E* (Equations 17, 18, 21, 22, 26) into the predictions for flowering time was substantial and improved on that observed in controlled environments. Of course, any direct comparisons need to be treated carefully here. The environment contrasts in controlled (day length) and field (rainfall and temperature) experiments are based on different underlying variables, which could potentially explain some differences, including the magnitude of improvement from including *G* × *E* under both the GBLUP and RHKS regression.

The transcriptome alone again performs better than *G* for predicting flowering time, though the relative improvement is not as large as seen in controlled environment experiments (**Table 1**). For height, there was no discernible advantage to including the transcriptome over SNP data alone, which underperformed relative to the model scenarios including *G* and *G* × *E* under the GBLUP framework. Inclusion of nonadditive effects (*G* × *E*, epistasis and dominance) under the GBLUP framework all improved model accuracy, counter to day length experiments. The improvement in prediction accuracy for both traits, particularly flowering time, through the inclusion of *G* × *E* remained substantial relative to epistasis and dominance effects. As with the controlled environments, RHKS outperformed the GBLUP equivalents. Finally, in contrast to controlled environments, the RHKS framework, for the best-performing models combining all data types, outperformed or performed equally well to the equivalent GBLUP model.

**Table 1:** Pearson correlation, as defined by Equation 27, was used to indicate the predictive accuracy of model performance from the controlled experiment data.

Controlled environment		
Model	Height (cm) (std)	Athesis (DAS) (std)
(1) *G*	0.789 (0.027)	0.275 (0.070)
(2) *G* + *G* # *G* (EPI)	0.785 (0.029)	0.268 (0.058)
(3) *G* + *G* # *G* +*A* (dominant)	0.782 (0.030)	0.253 (0.064)
(4) *G* + *G* × *E*	0.837 (0.025)	0.837 (0.011)
(5) *G* + *G* × *E* + *G* # *G*	0.836 (0.024)	0.834 (0.013)
(6) *G* + *T*	**0.840 (0.030)**	0.822 (0.016)
(7) *G* + *T* + *G* # *G*	0.838 (0.026)	0.819 (0.017)
(8) *G* + *T* + *G* × *E* + *G* # *G*	**0.840 (0.023)**	**0.843 (0.013)**
(9) *G* + *T* + *G* × *E* + *G* # *G* + *A*	0.836 (0.025)	0.839 (0.015)
(10) RHKS: *G* + *G* # *G* + *A*	0.784 (0.030)	0.240 (0.067)
(11) RHKS: *T*	0.836 (0.028)	0.811 (0.011)
(12) RHKS: *G* + *G* # *G* + *A* + *T*	0.836 (0.028)	0.811 (0.011)
(13) RHKS: *G* + *G* # *G* + *A* + *T* + *G* × *E*	0.838 (0.023)	0.837 (0.015)

The mean and standard deviation (std; shown in parentheses) of Pearson correlations were calculated from fivefold cross validation between training and test populations based on 13 different model scenarios described in Sect. 2.2.4.

Bolded values indicate the best models for the two traits of interest.

In summary, the most predictive models were those that combined all data types. In both sets of experiments, the transcriptome is predictive of both traits, but much more so in controlled environments. This is somewhat expected because gene expression better captures how genes contribute to trait development under different environments, as noted by Michel et al. (2021). The inclusion of the transcriptome (Equations 19-22, 24-26) potentially helps capture key genes and pathways involved in trait expression, which can refine predictive models. The nonlinear regression model did not show many advantages over the traditional linear mixed models from these two data sets, especially for the controlled environmental data. It outperformed or was similar to GBLUP in field conditions, possibly reflecting that in controlled environments, genotype and environment effects and interactions are less complex and more easily delineated, allowing for the isolation of these variables from more complex environmental interactions, with implications for suitability of different model frameworks. In both data sets, the site/treatment covariates simplify the environmental variation, which can reduce the impact of complex nonadditive genomic interactions that are more pronounced in variable natural conditions, as discussed by Teressa et al. (2021) and Becker and Leon (1988).

## 4 Discussion

Crop breeding is at a pivotal point, driven by the need to address challenges such as population growth, limited arable land, and environmental changes. To achieve profitable and sustainable crop production, the industry requires solutions that enable accurate, high-throughput decision-making to expedite the development of improved crop cultivars. This involves targeting staple crop genetics, environmental factors, and management practices. Modern statistical breeding technologies, including advanced methods for integrating multiple objectives into genetic evaluations, are fundamental for decision-making.

We examined the potential of the transcriptome as an alternative predictor in GS for wheat developmental traits, specifically flowering time and height. The use of transcriptomic data to enhance GS has gained attention in recent years (Xu et al., 2017). Studies have explored the transcriptome’s predictive value in crops such as maize (Zheng et al., 2017; Schrag et al., 2018; Azodi et al., 2019) and rice (Wang et al., 2019), but its application in wheat, a major global food crops, remains underexplored. Furthermore, most studies focus on controlled conditions, whereas applying omics data in real-world settings is crucial for assessing its utility in practical breeding programs.

We selected flowering time and plant height for this study because these traits are well-characterised, with extensive datasets, including transcriptomes, available to support testing of the framework. Importantly, this framework also holds value for selecting these key agronomic traits within wheat breeding programs. Although both traits are primarily regulated by major quantitative trait loci (QTL) and can be optimised in elite germplasm through early-stage phenotypic selection, there is additional genomic variation within elite backgrounds. This includes complex genetic loci and interactions that can be leveraged to enhance trait optimisation through genomic selection. For example, while the major dwarfing and semi-dwarfing alleles (RHT1 and RHT2) largely control variation in plant height, numerous additional QTL have been identified in recent studies (Shaheen et al., 2024). Regarding flowering time, VRN1 accounts for a substantial portion of variation under nonvernalising conditions. However, under vernalising conditions, smaller-effect loci—responsive to day length, drought, and heat—also influence flowering time (Hyles et al., 2020). When considering the epistatic interactions and genotype–environment (*G* × *E*) effects associated with these loci, GS becomes particularly advantageous because of its ability to capture these complex effects, enabling more accurate and effective selection. Consistent with previous findings, we found the transcriptome to be an effective phenotype predictor within both GBLUP and RHKS regression models, particularly outperforming genomic SNPs for anthesis and heading date and height in controlled conditions when the environment was excluded from the model.

The underperformance of genomic SNP models in predicting flowering time likely stems from the trait’s strong dependence on genotype–environment interactions, such as day length and temperature (Susila et al., 2018; Ausin et al., 2005). This effect may have been more pronounced under nonvernalising conditions in controlled environments, due to interactions between the VRN1 locus, which can delay flowering in vernalisation-sensitive lines, and photoperiod-sensitive genes (PPD1) and the FT locus under different day length conditions (Hyles et al., 2020). Excluding environmental factors as in model scenarios (Equations 14-16, 23), which considered only genetic factors in isolation, led to poor cross validation accuracy in genomic prediction. In contrast, when *G* × *E* were included, the model explained a much higher proportion of the variation in flowering time. The transcriptome’s improved performance suggests it captures these genotype–environment interactions intrinsically, making it a more reliable predictor of flowering time (see **Tables 1**, **2**). This aligns with the hypothesis that the transcriptome, as an intermediary between the genome, environment, and phenotype, effectively captures these effects and should be a reliable predictor of trait variation (Te Pas et al., 2017).

**Table 2:** Pearson correlation, as defined by Equation 27, was used to indicate the predictive accuracy of model performance from field experiment data.

Field experiments		
Model	Height (cm) (std)	Days to heading (std)
(1) *G*	0.533 (0.065)	0.245 (0.087)
(2) *G* + *G* # *G* (EPI)	0.539 (0.066)	0.247 (0.084)
(3) *G* + *G* # *G* + *A*	0.545 (0.076)	0.251 (0.073)
(4) *G* + *G* × *E*	0.644 (0.041)	0.925 (0.005)
(5) *G* + *G* × *E* + *G* # *G*	0.650 (0.044)	0.924 (0.004)
(6) *G* + *T*	0.625 (0.033)	0.602 (0.042)
(7) *G* + *T* + *G* # *G*	0.627 (0.036)	0.603 (0.041)
(8) *G* + *T* + *G* × *E* + *G* # *G*	0.656 (0.033)	**0.967 (0.009)**
(9) *G* + *T* + *G* × *E* + *G* # *G* + *A*	**0.660 (0.042)**	**0.966 (0.009)**
(10) RHKS: *G* + *G* # *G* + *A*	0.548 (0.072)	0.253 (0.095)
(11) RHKS: *T*	0.536 (0.030)	0.591 (0.044)
(12) RHKS: *G* + *G* # *G* + *A* + *T*	0.558 (0.134)	0.566 (0.058)
(13) RHKS: *G* + *G* # *G* + *A* + *T* + *G* × *E*	**0.662 (0.044)**	**0.966 (0.015)**

The mean and standard deviation (std; shown in parentheses) of Pearson correlations were calculated from fivefold cross validation between training and test populations based on 13 different model scenarios described in Sect. 2.2.4.

Bolded values indicate the best models for the two traits of interest.

In the case of height, we observe a different pattern. While incorporating *G* × *E* interactions enhances model predictive accuracy in both controlled conditions and field settings, the relative gain is less pronounced compared to flowering time. This suggests that the simpler genetics underlying height, primarily major genes from the Rht family (Zheng et al., 2017; Achard et al., 2009), exhibit fewer environmental interactions in these experiments. Notably, in the field, using transcriptome data did not offer an advantage over genomic SNPs. This could be explained by the fact that these key height-controlling genes are not expressed until later in development (Borrill et al., 2022); hence transcriptomes collected at the earlier stage in this study would be unlikely to capture the *G* × *E* effects at these loci.

Although the transcriptome offers predictive benefits for flowering time, particularly in controlled environments, its effectiveness in field conditions is reduced. This likely reflects the incompleteness with which a transcriptome taken at a single time point early in development can capture the *G* × *E* effects experienced throughout development to maturity in the field. The controlled environment experiments would not suffer this limitation, as environmental conditions were maintained throughout development, preserving the relationship between the regulatory signal captured in the early-stage transcriptome and the trait. This highlights a challenge in using highly plastic omics data, such as the transcriptome, for GS in variable environments, where multiple tissues and time points might be needed to capture relevant interactions, reducing its feasibility in commercial breeding programs. Nonetheless, it is notable that the early-stage transcriptome offers some predictive power for flowering time in field conditions.

Our findings indicate that while incorporating the transcriptome or directly modelling genotype–environment interactions is essential for reliable predictions, especially for flowering time, other nonadditive factors such as epistasis and dominance made only minor contributions. Consistent with expectations (Gianola and Van Kaam, 2008), RHKS was slightly better at capturing complex genomic random effects compared to the GBLUP model (e.g., model scenarios 13 and 9; 10 and 3), although the advantage was marginal.

The best-performing models for both traits combined all data types, genotype–environment interactions, and epistasis under the GBLUP framework in controlled conditions and the RHKS framework in the field. The RHKS model’s marginal advantage in field conditions may reflect the greater complexity of environmental variables that the nonlinear Gaussian kernel can better capture, e.g., Cuevas et al. (2017). However, in controlled environments, where conditions are simplified and *G* × *E* interactions are likely less complex, the GBLUP model is promising. Despite the predictive advantages of the transcriptome, the high cost and complexity of incorporating it into breeding programs currently limit its practicality. Additionally, the need to structure sampling around developmental and environmental cues for effective trait prediction adds another layer of complexity, which affects the utility of omics data for GS in commercial breeding programs. For the time being, the strength of population-scale transcriptomics lies in enhancing our biological understanding of complex genomic interactions, which can then be integrated into breeding selection models in alternative ways (Khalilisamani et al., 2024).

This study demonstrates a proof of concept for integrating genomic, multi-omic, environmental, and phenotypic data into an advanced statistical analytic framework to improve genomic prediction in wheat breeding. In both controlled and field experiments, transcript data perform well relative to SNPs or environmental data alone in predicting plant height and flowering time. This is likely due to the transcriptome’s ability to capture both genetic and environmental signals and their interactions, thereby enhancing the effectiveness of GS. By integrating the transcriptome with genetic SNPs and *G* × *E* interactions, our models provide a highly accurate and comprehensive solution to predict both flowering time and height. In both the GBLUP and RHKS frameworks, the models integrating SNPs and *G* × *E* interactions outperformed the transcriptome-based predictions, including all three types of predictors (SNPs, transcriptome, and *G* × *E*) provides only a marginal gain in predictive accuracy. Given the practical and cost-related constraints of implementing transcriptome data in GS, incorporating SNP and *G* × *E* effects remains a more feasible approach, provided that environmental factors can be accurately characterised. Recent studies (Varona et al., 2018; Morais Junior et al., 2017) support this, showing that accounting for *G* × *E* interactions improves suitability and prediction accuracy, allowing for more reliable genetic evaluation in diverse environments. Breeders can thus identify the performance of genotypes for targeted traits and their adaptation across multiple environments.

The statistical framework we present is agnostic regarding the crop and trait, providing flexibility for predicting genetic merit in various scenarios and identifying variations in multiple data streams for any target trait, thereby reducing uncertainty in GS and accelerating the development of new wheat varieties. However, effectively scaling this interaction model to large-scale datasets in breeding programs may become infeasible. For example, complex traits such as yield and disease resistance are influenced by a variety of environmental factors, e.g., soil, weather, and pathogens. Incorporating multiple environmental factors with real value is essential for analysing *G* × *E* interactions (Smith et al., 2021), which requires further development to improve *G* × *E* modelling framework and computational efficiency in handling large-scale crop data. Furthermore, complex traits arise from the interaction of multiple genetic factors, resulting in different weights of marker data associated with their QTL. Multiple traits can also work together to influence a single gene. To enhance genomic predictive accuracy in wheat breeding, it may be necessary to apply marker-weighted techniques (Montesinos-López et al., 2023) and develop multi-trait interaction modelling (Mardia et al., 2024).

## Data Availability

The original contributions presented in the study are publicly available. This data can be found here: https://doi.org/10.25919/vxt2-0042.
